# Knowledge, Attitudes, and Practices Survey among Nursing Care Workers Involved in Caring for Older Adults during the Early Stage of the COVID-19 Pandemic in Japan

**DOI:** 10.3390/ijerph192012993

**Published:** 2022-10-11

**Authors:** Dan Kambayashi, Toshie Manabe, Masayoshi Hirohara, Hiroyasu Akatsu

**Affiliations:** 1 Laboratory of Pharmacy Practice, Center for Education and Research on Clinical Pharmacy, Showa Pharmaceutical University, Tokyo 194-8543, Japan; 2 Department of Medical Innovation, Nagoya City University Graduate School of Medicine, Nagoya 467-8601, Japan; 3 Center for Clinical Research, Nagoya City University West Medical Center, Nagoya 462-8508, Japan; 4 Department of Community-Based Medical Education, Nagoya City University Graduate School of Medicine, Nagoya 467-8601, Japan; 5 Fukushimura Hospital, Toyohashi 441-8124, Japan

**Keywords:** COVID-19, knowledge attitudes practices survey, nursing care workers, older adults

## Abstract

Background: As Japan undergoes population aging, nursing care workers play an important role in supporting older adults in the community, which has been particularly critical during COVID-19 pandemic. However, the knowledge, attitudes, and practices (KAP) among nursing care workers regarding COVID-19 have not been fully elucidated. Methods: A self-administered questionnaire survey was conducted in June 2020 among 481 nursing care workers in the nursing care facilities in Aichi, Japan. We assessed COVID-19-related KAP scores of nursing care workers, and compared them by age, sex, and years of experience. Results: A total of 481 nursing care workers responded to the survey. Out of a maximum of 10 points, the mean (standard deviations) knowledge, attitude, and practice scores were 6.86 (1.45), 7.11 (1.42), and 7.40 (1.89), respectively. Comparisons between the KAP scores revealed significantly higher knowledge scores among older workers (*p* < 0.001) and significantly higher knowledge scores (*p* = 0.002) and practice scores (*p* = 0.033) among workers with more than 20 years of working experience. Conclusions: The findings revealed that older age and a longer duration of experience were associated with higher COVID-19-related knowledge and practice scores. To better support older adults, it is essential to improve the education system for care workers and to provide environments for delivering necessary information rapidly.

## 1. Introduction

As the coronavirus disease 2019 (COVID-19) continues to spread around the world, public health crisis prevention has involved the adoption of behavioral restrictions in daily life to avoid infection [1]. However, the unintended negative effects of this behavior change have included decreased physical activity and increased mental distress [2], in addition to the deterioration of mental health among children and adolescents [3]. Various secondary health effects of the COVID-19 pandemic have been observed in all generations living in the community. Among these effects, decreased physical activity in older adults has been reported in a systematic review [4] and is directly related to worsening frailty. In addition, frailty has been reported to be associated with mortality risks [5].

In Japan’s aging society, in which 28.7% of the population in 2020 was older than 65 years [6], care workers at nursing homes are among the professionals with whom older adults most often interact. Japanese nursing care workers practice care that supports dignity and independence while interacting with older adults. These workers not only provide physical support but also psychological and social support. Regardless of whether an older adult lives in a facility or at home, this psychological and social care helps to support older adults’ quality of life. Nursing care workers are responsible for assisting with various aspects of the daily lives of older adults. This has included providing information about COVID-19 prevention methods and infection-related problems. It is also important to consider ways of improving COVID-19 knowledge and creating a place and framework [7] within which to do this. Thus, understanding the COVID-19-related knowledge, attitudes, and practices (KAP) of nursing care workers is crucial for ensuring the quality of life of older adults. This understanding may also be useful for developing COVID-19 countermeasures. However, to the best of our knowledge, no previous studies have investigated COVID-19 KAP among nursing care workers in Japan [8].

The present study examined whether nursing care workers in Japan had sufficient levels of KAP regarding COVID-19 for taking care of older adults during the pandemic. In the early stages of the pandemic in Japan, approximately 1200 new laboratory-confirmed cases were reported per week, with approximately 19,000 cumulative cases. We sought to determine whether nursing care workers had sufficient KAP to assist in reducing the risks of infection and infection-related problems for older adults in nursing and healthcare facilities under the conditions of the COVID-19 pandemic.

## 2. Materials and Methods

### 2.1. Study Design, Study Site, Participants, and Data Collection

This cross-sectional study used a self-administered questionnaire survey. The participants were nursing care workers who worked for a social welfare corporation, consisting of nursing homes, a geriatric health services facility, and a support facility for disabled older adults in Aichi, Japan, at the time of the study. The study site contained 550 nursing care workers. Based on previous studies [9,10], we calculated the required sample size for this study as 227 participants, with a total population of 550 persons, a confidence level of 95%, an error of 5%, and a response distribution of 50%. We recruited participants using posters at the facilities. To protect participants’ personal information and to avoid sharing question information, participants posted the questionnaire in a designated box installed in a facility that could only be accessed by specific researchers after completing the questionnaire.

The study was approved by the ethical review committee of Showa Pharmaceutical University (Approval No. 2020-12) and by the institutional review board of Choju Medical Institute, Fukushimura Hospital (Approval No. 419). Written informed consent was obtained from the study participants.

### 2.2. KAP Survey Methodology

We conducted a KAP survey from 1 June to 30 June 2020 using a self-administered questionnaire. The questionnaire was designed by the study investigators based on “Risk communication and community engagement action plan guidance for COVID-19 preparedness and response” [11], “A guide to developing knowledge, attitude, and practice surveys” [12] published by the World Health Organization, and on previous related studies [9,10,11,12,13,14,15] (Questionnaire S1). The questionnaire was designed to assess COVID-19-related KAPs. In addition, we collected information about participant characteristics, including age, sex, nationality, academic background, number of people in household, and underlying diseases. The questions were either multiple choice or closed-ended, and participants chose their responses from a provided set of answers (Yes/No, True/False, or Agree/Disagree/Undecided). The questions were scored using a 3-point Likert scale: 3 points = “agree”, representing a positive attitude; 2 points = “undecided”; 1 point = “disagree”. In the case of multiple choice questions, 1 point was assigned for each correct choice. For knowledge-related responses, cutoff points were set according to the number of correct answers, with 0 to 3 points assigned according to the number of correct answers [16,17]. For responses regarding practice, 1 point was given depending on the respondents showing correct practice behavior. The KAP scores were calculated from the answers using factor analysis, adjusted to give a total score of 10 points being the highest possible score [10]. The KAP scores were compared by demographic group (age, sex, and years of working experience). We conducted a pilot study using a preliminary questionnaire and confirmed that there were no problems with it. The Cronbach’s alpha coefficient for internal consistency in the KAP scores was 0.747.

### 2.3. Statistical Analysis

The collected data were expressed as the mean and standard deviation (SD) or the median and interquartile range (IQR) for continuous variables. Comparisons between groups were made using Student’s *t*-test or the Kruskal–Wallis test for continuous variables and the χ^2^ test or Fisher’s exact test for categorical variables. If any variables were missing from participants’ responses, we reduced the number of participants and performed another comparison of the groups. For all analyses, the significance levels were two-tailed and *p* < 0.05 was considered to indicate statistical significance. The analyses were carried out using IBM SPSS, version 28.0 (IBM Corp., Armonk, NY, USA).

## 3. Results

### 3.1. General Characteristics of the Participants

Of the 538 nursing care workers recruited from the social welfare corporation facilities, 481 (89.4%) agreed to participate in the study and completed the self-administered questionnaire. The general characteristics of participants are shown in Table 1.

Of the 481 participants, the median age was 45 years and the median number of years of experience as a nursing care worker was 7 years. Women accounted for 74.4% of participants. The academic background of the participants was mainly high school (48.6%) and most participants had no comorbidities (76.3%).

### 3.2. Knowledge Relating to COVID-19 among the Nursing Care Workers

The results relating to COVID-19 knowledge are shown in Table 2.

In terms of COVID-19-related knowledge, 74.8% of participants had knowledge about “symptoms of COVID-19 infection”, 69.8% had knowledge about the occurrence of “asymptomatic patients”, and 68.3% had knowledge about “how to respond to prevent infection”. Almost all participants (97.1%) knew that the disease is transmitted by droplets from coughing and sneezing, but a lower proportion had knowledge about the risk of death (37.9%) or the medical care system required for COVID-19 patients (18.9%). Only 9.6% of participants felt that they had sufficient information about COVID-19. The most commonly reported COVID-19 symptoms were fever (96.7%), breathlessness (92.3%), cough (82.7%), and fatigue (81.6%). Less than half of the participants answered that myalgia, headache, and diarrhea could be symptoms.

### 3.3. Information Resources Regarding COVID-19 among Nursing Care Workers

**Figure 1:**
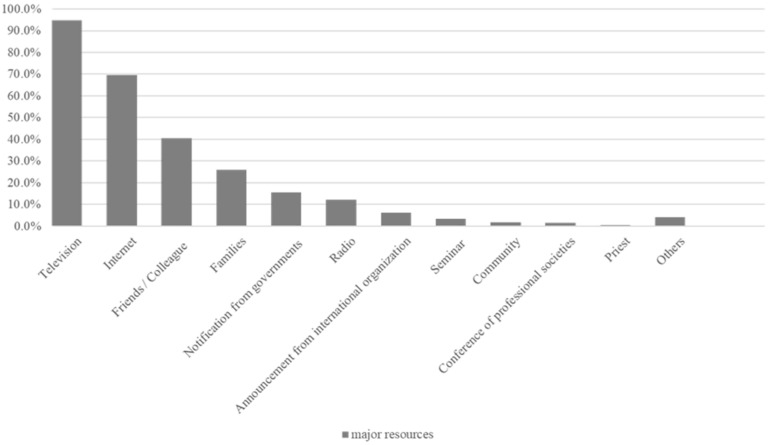
Information resources regarding COVID-19 among nursing care workers.

The primary sources of COVID-19 information used by participants are presented in Figure 1. 

The main resources of COVID-19-related information among the nursing care workers were television (94.8%), the internet (69.6%), and friends/colleagues (40.4%), while announcements from international organizations (6.3%) and academic conferences (1.5%) were less often used.

The results of a two-group comparison by age (45 years or older vs. 44 years or younger) regarding COVID-19-related information resources are shown in Figure S1. Compared with the younger age group, the older age group was significantly more likely to use government notices as a resource (19.9%, *p* = 0.002). However, television (96.5%, *p* = 0.050) was used by a proportion of participants in both groups. The younger age group was significantly more likely than the older group to use the internet (81.3%, *p* < 0.001) and family (31.8%, *p* = 0.012) as resources of information. A comparison by years of nursing care experience indicated that the group with more years of experience contained significantly more participants who used television (98.2%, *p* = 0.004) as a resource of information compared with the group with less experience (Figure S2).

### 3.4. Attitudes Relating to COVID-19 among Nursing Care Workers

The results relating to COVID-19 attitudes are shown in Table 3.

Overall, 62.7% of participants felt that their occupation as nursing care workers put them “at high risk of infection with COVID-19”. The reasons for feeling threatened by COVID-19 included “I might be infected” (84.7%) and “I might pass it on to someone else” (83.9%).

The percentage of respondents who felt embarrassed about being infected with COVID-19 was 39.1%. Despite the high risk of COVID-19 infection, 85.8% of the participants wanted to keep their current jobs and 89.1% were proud of their jobs. Among the participants, 72.1% thought that influenza was a preventable disease, but only 38.7% thought that COVID-19 was preventable. The attitude questions revealed that most of the nursing care workers were fearful of becoming infected with COVID-19; however, they were proud to continue working.

**Figure 2:**
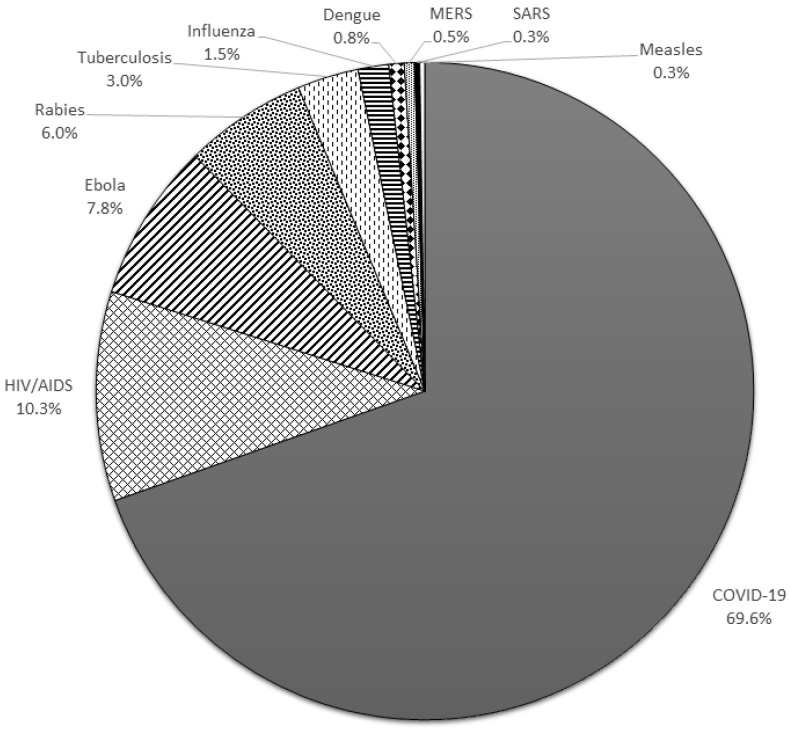
Most-feared infectious diseases among nursing care workers.

When asked which infectious disease was the most frightening, the most common responses were COVID-19 (69.6%), HIV/AIDS (10.3%), and Ebola (7.8%) (Figure 2).

### 3.5. Practices Relating to COVID-19 among Nursing Care Workers

The results relating to COVID-19 practices are shown in Table 4.

The event that most commonly influenced self-restraint behaviors was the “declaration of a state of emergency by the government” (79.7%), followed by the “death of a celebrity” (52.4%), and “news media about the infection of famous people” (30.4%).

The most common actions taken by the respondents or their family members when they suspected COVID-19 infection were “call the health center for advice” (87.4%) and “contact the workplace” (80.8%). Regarding COVID-19 infection control measures, “wearing a mask when around other people”, “hand hygiene”, and “ventilate the room regularly” were implemented by more than 90% of the participants. Infection control measures implemented in the workplace were “ventilate the room regularly” (92.0%), “disinfecting indoor items and equipment” (71.4%), and “alerting and educating patients about COVID-19” (24.2%). These results revealed that basic infection control measures were widely practiced by the respondents themselves and in their workplaces, but fewer education and interventions were given to the older adults.

### 3.6. Differences in COVID-19-Related KAP Scores among Demographic Groups

We calculated COVID-19-related KAP scores based on the responses to the questionnaire items and assessed them by demographic group (Table 5).

Out of the maximum attainable scores of 10 points, the mean (standard deviations) knowledge, attitude, and practice scores of nursing care workers in the early stages of the COVID-19 pandemic were, 6.86 (1.45), 7.11 (1.42), and 7.40 (1.89), respectively. The COVID-19 knowledge score was significantly higher in the older group (45 years and older) than in the younger group and was the highest in the group with 20 years or more of experience (*p* < 0.001 and *p* = 0.002, respectively). There were no significant differences in attitude scores among the groups, but the practice score was highest in the group with 20 years or more of experience (*p* = 0.033).

## 4. Discussion

This study was conducted in the early stages of the COVID-19 pandemic and indicated that nursing care workers in Japan generally had a basic knowledge of COVID-19. Additionally, as age and years of experience increased, COVID-19-related KAP scores also increased. These results suggest that nursing care workers with a basic knowledge of COVID-19 can help patients avoid infection and are familiar sources of information for older adults, and that nursing care workers with extensive practical experience may play a particularly important role in infection prevention.

Nursing care workers work closely with older adults, assisting them with exercise, eating, and bathing. They also engage older adults in conversation about various topics to prevent cognitive decline. In this way, nursing care workers are essential workers who are instrumental in the lives and health of older adults living in facilities. In the care sector, as in the medical sector, it is not possible to completely avoid being in close proximity to other people and it is not possible to carry out the necessary tasks via telework. Therefore, nursing care workers must take care to not become infected with COVID-19 and to not pass it on to their patients. Despite the fear surrounding COVID-19 and the anxiety regarding being infected without showing symptoms, these workers continued to collaborate with their colleagues to support their patients and their patients’ families and to be comfortable and happy.

The results of the knowledge questionnaire revealed that the workers had a general and basic knowledge of COVID-19. However, only 9.6% of participants reported that they had sufficient knowledge of COVID-19. The main sources of COVID-19 information were television and the internet, with considerably less information obtained from international organizations and academic conferences. These results are similar to those of a survey conducted in Japan regarding community pharmacists’ COVID-19 knowledge, in which the main resources of information were the internet and television, with less use of academic conferences and other resources [16]. The COVID-19 pandemic has led to a flood of information being disseminated via the internet. Misinformation has been found to have a substantial influence on decision making [17,18], and there is evidence that it can contribute to mental disorders and other health problems [19]. To avoid the negative outcomes caused by misinformation, we believe that it is necessary to develop a system to provide accurate and up-to-date information regarding COVID-19 for care workers and an environment in which to implement educational programs on emerging infectious diseases. The current results revealed that more experienced and older nursing care workers were more likely to watch television and be aware of government notices, which suggests that they actively seek information about COVID-19 (Figures S1 and S2). Because we conducted the present study in the early stages of the pandemic, the rate of attendance of COVID-19-related conferences was low among the participants. Therefore, conference attendance was not reflected in participants’ KAP scores. However, if they have additional opportunities to participate in seminars, increasing these opportunities may help them to improve their knowledge and to provide better care to older adults. Knowledge scores were higher for nursing care workers who were older and those who were more experienced, indicating that information and means of easy access for older adults may affect the scores. A previous study that examined the effect of educational intervention among residents regarding avian influenza H5N1 infection in Vietnam showed an increase in “healthcare workers” as an information resource after the educational program provided by the healthcare workers [9]. Although it has been suggested that increasing knowledge about COVID-19 is important for the nursing care workers themselves, it is also useful for increasing knowledge and appropriate behaviors for preventing infection among older adults who are receiving care from the nursing care workers. In addition, at the time of the influenza H1N1pdm09 pandemic, a study in Mexico indicated that one of the risk factors of hospitalization was a lack of information regarding the importance of early access to health care [20]. The Mexican government has used taxi windows to distribute information and to encourage people to seek medical care early and to announce that there was a free medical care program. Thus, it is crucial to consider information resources, to ensure that people receive the necessary information about unfamiliar issues, such as emerging infectious diseases.

The attitude-related results indicated that nursing care workers felt threatened by COVID-19 and were concerned not only about becoming infected but also about infecting others. They were also aware that their job entailed a high risk of infection, yet they wanted to continue to carry out their responsibilities with pride. It is reassuring for older adults living in the community and in nursing care facilities to have access to dedicated and reliable nursing care workers. The provision of appropriate advice to older adults by care workers is valuable for reducing the risk of COVID-19 infection and related problems.

Of all infectious diseases, nursing care workers stated that COVID-19 was the one they feared most. This may have been because of the uncertainty in the early stages of the pandemic when little was known about the virus and understanding was limited. In addition, a substantial proportion of respondents selected rabies as their most-feared infectious disease, which may have been because rabies was still being reported in the district at the time of the survey.

In Japan, the government’s declaration of a state of emergency strongly influenced people’s behaviors, but it seems to have gradually lost its perceived importance because a state of emergency has been declared many times. In contrast, in Sweden, which is based on the autonomy of the people in Europe, a high degree of trust in the government and a high degree of adherence to infection control measures have been reported [21]. This may be because of differences in the cultural context and the infection situation. To change people’s behavior in response to COVID-19, it is essential that they have accurate knowledge that is based on scientific information and the ability to understand, accept, and act on this information. In terms of the impact on personal behaviors, significantly more nursing care workers in the older age group cited the “death of a celebrity” and “reports of infectious diseases in celebrities” as influencing factors. Such reports may represent fear mongering, which could prevent people from recognizing the true risk and acting appropriately. Misinformation about COVID-19 quickly spread through social media, causing confusion and fear, which made it difficult for people to deal with the pandemic [22].

Many nursing care workers were familiar with basic infection control practices for themselves and their facilities; however, they were less involved in alerting and educating others about COVID-19. This suggests the importance of education and shows that there is a need for an education program and an environment in which care workers can obtain the necessary knowledge. A meta-analysis of COVID-19-related KAP, which evaluated KAP in approximately 200,000 members of the general population, also revealed that education affected practice scores and that “knowledge and attitudes” also affected practice [8]. A systematic review reported that educational interventions for healthcare workers implemented in response to severe acute respiratory syndrome, Middle East respiratory syndrome, Ebola virus, and COVID-19 improved care worker attitudes and improved their knowledge and skills, indicating that these interventions were effective [23]. The educational interventions presented in the studies were related to training during a viral epidemic. Although the interventions varied in content, design, and mode of delivery, we believe that such educational strategies could be useful for nursing care workers who have frequent contact with older adults. Educational interventions among healthcare workers have led to improved satisfaction and experience [24,25,26], modification of learner attitudes, and increased confidence [27]. Additionally, there are reports of reduced infection rates among healthcare workers [28] and reduced mortality rates among extracorporeal membrane oxygenation patients [29] when healthcare workers participate in training programs. Thus, programs to support the education of healthcare professionals have a variety of positive effects. To be adequately prepared for the next emerging infectious disease pandemic, it is necessary to develop and implement evidence-based training programs that are easily adaptable to nursing care workers. Nursing care workers in Japan’s super-aging society need to improve their knowledge of COVID-19 and need to contribute to the care of the older adults. At the same time, the current conditions may constitute an opportunity for nursing care workers to consider a new place for knowledge creation [7], including what kind of knowledge is required for nursing care workers to properly care for the older adults during the COVID-19 pandemic.

The present study involved several limitations. The participating nursing care workers all worked at one institution; therefore, their responses may differ from those of nursing care workers nationwide. Because this study was conducted during the early stage of the COVID-19 pandemic, the results may differ from current KAP scores among nursing care workers. However, the relationship between nursing care workers and older adults has not changed, even after the pandemic has passed, and this will be valuable information for developing countermeasures against infection among older adults in the current stage of the COVID-19 pandemic. Furthermore, the results of this study will serve as basic data for countermeasures against older adults in the early stages of the pandemic and will be useful for countermeasures against emerging infectious diseases that may occur again in the future.

## 5. Conclusions

Nursing care workers had high levels of knowledge, attitudes, and practices regarding COVID-19. The current results revealed that, as the age and experience of nursing care workers increased, their COVID-19-related knowledge and practice scores also increased. The findings also suggested that these workers play an important role in supporting the health of older adults. Providing accurate and timely information and education to nursing care workers will be important for supporting older adults and for reducing the risk of infection among this vulnerable population. Sharing the experiences of nursing care workers in the super-aging society of Japan with those in other countries is crucial in the era of the COVID-19 pandemic.

## Figures and Tables

**Table 1:** Participants’ characteristics.

	Participants, *n* (%) (*n* = 481)
Demographic information	
Age, median (IQR)	45 (35–53)
Sex, *n* (%)	
Male	123 (25.6)
Female	358 (74.4)
Nationality, *n* (%)	
Japanese	428 (89.4)
Non-Japanese	51 (10.6)
Academic background, *n* (%), *n* = 475	
Junior high school	20 (4.2)
High school	234 (49.3)
Junior college/Technical school	115 (24.2)
University	89 (18.7)
Graduate school	14 (2.9)
Number of people in household, *n* (%)	
Living alone	77 (16.2)
2	110 (23.2)
3	96 (20.2)
4	103 (21.7)
5	58 (12.2)
≥6	31 (6.5)
Underlying disease *, *n* (%), *n* = 467	
None	367 (78.6)
Cerebral infarction	4 (0.9)
Angina/Arrhythmia	9 (1.9)
Hypertension	31 (6.6)
Diabetes	21 (4.5)
COPD/Asthma	6 (1.3)
Others	42 (9.0)
Years of experiences as a nursing care worker, median (IQR) (*n* = 425)	7 (3–13)
Have you ever attend a seminar or conference regarding COVID-19?—Yes (%), *n* = 478	35 (7.3)
Have you ever cared for a patient with COVID-19?—Yes, *n* = 479	1 (0.2)
Have you ever been infected with COVID-19?—Yes	2 (0.4)

Abbreviations: IQR—interquartile range; COPD—chronic obstructive pulmonary disease. * Multiple choice question.

**Table 2:** Knowledge relating to COVID-19 among nursing care workers.

	Participants, *n* (%) (*n* = 481)
Having enough information about COVID-19, *n* (%)	46 (9.6)
What do you know about COVID-19 *, *n* (%), *n* = 477	
Do not know anything about COVID-19	27 (5.7)
Infection protection measures	326 (68.3)
Symptoms of infected people	357 (74.8)
Route of infection	189 (39.6)
Actions to be taken at the onset of infection	286 (60.0)
Asymptomatic cases in COVID-19	333 (69.8)
Risks of severity	266 (55.8)
Required medical care system for COVID-19 patients	90 (18.9)
Risks for mortality	181 (37.9)
Infect by droplets such as coughing and sneezing, *n* = 479	465 (97.1)
There are no antiviral drugs that are effective against COVID-19, *n* (%), *n* = 480	201 (41.9)
There are no vaccines against COVID-19, *n* (%), *n* = 480	303 (63.1)
Is this the proper way as the prevention measure against COVID-19? *, *n* = 478	
Avoid being bitten by mosquitoes	24 (5.0)
Hand wash using soap and running water	436 (91.4)
Wear a mask where there are people	453 (94.8)
Avoid direct contact with a person with high fever	411 (86.0)
Ventilate the room	455 (95.2)
Stay in the room as much as possible	150 (31.4)
Eat well-balanced meals	319 (66.7)
Eat meals alone	58 (12.1)
Sleep well	378 (79.1)
Is this the major symptom of COVID-19? *, *n* (%) *n* = 479	
Fever	463 (96.7)
Cough	396 (82.7)
Breathlessness	442 (92.3)
Myalgia	140 (29.2)
Headache	212 (44.3)
Diarrhea	106 (22.1)
Fatigue	359 (74.9)

* Multiple choice question.

**Table 3:** Attitudes relating to COVID-19 among nursing care workers.

	Participants, *n* (%) (*n* = 481)
My job holds a high risk against COVID-19 infection, *n* (%), *n* = 480	
Agree	301 (62.7)
Disagree	57 (11.9)
Neither agree/disagree	122 (25.4)
I feel a possibility of infection, *n* (%) *n* = 456	405 (88.8)
How threatened do you feel by COVID-19? *n* (%), *n* = 468	
Very threatened	310 (66.2)
Threatened	145 (31.0)
Not so threatened	13 (2.8)
What is the cause of feeling threatened by COVID-19? *, *n* (%), *n* = 478	
I may infect	405 (84.7)
I may let someone infect me	401 (83.9)
I may die	282 (59.0)
I may be quarantined if infected	189 (39.5)
My work and daily life may be limited	309 (64.6)
Currently, there are no effective medications or vaccinations	304 (63.6)
I will feel embarrassed if I become infected with COVID-19, *n* (%) *n* = 450	176 (39.1)
I think COVID-19 will converge, *n* (%)	
Agree	131 (27.2)
Disagree	98 (20.4)
Neither agree/disagree	252 (52.4)
The infection control measures in the workplace where I work are sufficient, *n* (%)	
Agree	212 (44.1)
Disagree	54 (11.2)
Neither agree/disagree	215 (44.7)
I want to keep my current job despite the high risk of COVID-19 infection, *n* (%) *n* = 445	382 (85.8)
I am proud of my job, *n* (%) *n* = 451	402 (89.1)
COVID-19 is a preventable disease, *n* (%) *n* = 476	
Agree	184 (38.7)
Disagree	41 (8.6)
Neither agree/disagree	251 (52.7)
Influenza is a preventable disease, *n* (%) *n* = 476	
Agree	343 (72.1)
Disagree	41 (8.6)
Neither agree/disagree	92 (19.3)

* Multiple choice question.

**Table 4:** Practices relating to COVID-19 among nursing care workers.

	Participants, *n* (%) (*n* = 481)
What was the issue that affected your preventable behavior? *, *n* (%), *n* = 473	
State of emergency by the government	377 (79.7)
Deceleration of pandemic by WHO	116 (24.5)
Face of the news for death of celebrities	248 (52.4)
Infection of close person	15 (3.2)
Death of close person due to COVID-19	8 (1.7)
Media news about the infection for famous people	144 (30.4)
What do you do if you or a family member has symptoms that suggest COVID-19 infection *, *n* (%) *n* = 475	
Seek medical care immediately	192 (40.4)
Call the health center for advice	415 (87.4)
Contact the workplace	384 (80.8)
Talk to family	261 (54.9)
Talk to a friend or colleague	92 (19.4)
Consult the family doctor	180 (37.9)
Go to the pharmacy and consult a pharmacist	4 (0.8)
Do not tell anyone	8 (1.7)
Do not go out from the house	294 (61.9)
Infection measures I actually do for COVID-19 *, *n* (%) *n* = 480	
Wear a mask where there are other people	475 (99.0)
Hand hygiene	455 (94.8)
Gargle	366 (76.3)
Ventilate the room regularly	435 (90.6)
Room cleaning and disinfection	276 (57.5)
Stay home on holidays	367 (76.5)
Avoid contact with people as much as possible	314 (65.4)
Eat well-balanced meals	264 (55.0)
Sleep well	293 (61.0)
Do nothing	1 (0.2)
Infection control measures that you implemented in the workplace *, *n* (%) *n* = 476	
Disinfect indoor items and equipment	340 (71.4)
Ventilate the room regularly	438 (92.0)
Alert and educate patients about COVID-19	115 (24.2)

* Multiple choice question.

**Table 5:** Differences in COVID-19-related knowledge, attitudes, and practices scores among nursing care worker demographic groups.

*n* = 389	Group	No. of Sample	Knowledge Score			Attitude Score			Practice Score		
			Score * (SD)	*p* Value	95% CI of Mean Difference	Score * (SD)	*p* Value	95% CI of Mean Difference	Score * (SD)	*p* Value	95% CI of Mean Difference
Total		389	6.86 (1.45)			7.11 (1.42)			7.40 (1.89)		
Age ^†^	≤44	171	6.54 (1.55)	<0.001	−0.863–−0.291	7.13 (1.46)	0.820	−0.252–0.318	7.21 (2.02)	0.073	−0.736–0.033
	≥45	218	7.11 (1.32)			7.09 (1.39)			7.56 (1.77)		
Sex ^†^	Male	106	5.85 (1.41)	0.968	−0.332–0.318	7.12 (1.58)	0.924	−0.325–0.359	7.24 (1.82)	0.282	−0.654–−0.191
	Female	283	6.86 (1.47)			7.10 (1.36)			7.47 (1.91)		
Years of working experience ^‡^	≤5 years	145	6.64 (1.59)	0.002		7.11 (1.45)	0.600		7.22 (2.10)	0.033	
	6–19 years	179	6.94 (1.30)			7.07 (1.46)			7.34 (1.73)		
	≥20 years	25	7.64 (1.35)			7.39 (1.22)			8.31 (1.37)		

Abbreviations: CI—confidence interval; SD—standard deviation. * A total score of 10 points being the highest possible score. ^†^ Comparisons between groups were made using Student’s *t*-test. ^‡^ Comparisons between groups were made using the Kruskal–Wallis test.

## Data Availability

All relevant data are within the manuscript and its Supporting Information files (Dataset S1).

## Supplementary Materials

The following supporting information can be downloaded at: https://www.mdpi.com/article/10.3390/ijerph192012993/s1, Figure S1: Resources of COVID-19-related information among nursing care workers by age group; Figure S2: Resources of COVID-19-related information among nursing care workers by years of work experience; Questionnaire S1: Questionnaire used in the study; Dataset S1: Dataset for the study.
