# Supplementary material for: Knowledge, Attitudes, and Practices Survey among Nursing Care Workers Involved in Caring for Older Adults during the Early Stage of the COVID-19 Pandemic in Japan
Source: Int J Environ Res Public Health. 2022 Oct 11;19(20):12993. doi: 10.3390/ijerph192012993 (PMC9602712; doi:10.3390/ijerph192012993)
Supplement: Supplementary file 1 [file ijerph-19-12993-s001.zip › QUESTIONNAIRE_S1.pdf]

Questionnaire

Q1 age

Q2 sex

1. male
2. female

Q3 nationality

1. Japanese
2. non-Japanese

Q4 Academic background

1. elementary school
2. Junior high school
3. High school
4. Junior college / Technical school
5. University
6. Graduate school

Q5 Number of people in household

1. Alone
2. 2 people
3. 3 people
4. 4 people
5. 5 people
6. ≥ 6 people

Q6 Years of experience as a nursing care worker

Q7 Do you have an underlying disease?

1. None
2. Cerebral infraction
3. Hypertension
4. Angina / Arrhythmia
5. Diabetes
6. COPD / Asthma

7. Others

Q8 Participated in academic societies, workshops, and study sessions about COVID-19.

1. Yes
2. No

Q9 Involved in the treatment and care of patients with COVID-19

1. Yes
2. No

Q10 Have you ever infected with COVID-19?

1. Yes
2. No
3. Do not know

Q11 Have enough information about COVID-19？

1. Yes
2. No
3. Do not know

Q12 What I know about COVID-19.

1. Do not know anything about COVID-19
2. Infection protection measures
3. Symptoms of infected people
4. Route of infection
5. Actions to be taken at the onset of infection
6. Asymptomatic cases in COVID-19
7. Risks of aggravation
8. Required medical care system for COVID-19 patients
9. Risks for mortality

Q13 Main resources for COVID-19 information？

1. Television
2. Radio
3. Internet
4. Family

5. Friends / Colleagues
6. Seminars
7. Conference of professional societies
8. Governmental notices
9. Announcements of international organizations
10. Priests
11. Community
12. Others

Q14 My job holds a high risk against COVID-19 infection

1. Agree
2. Disagree
3. Neither agree/disagree

Q15 I feel a possibility of infection.

1. Yes
2. No

Q16 COVID-19 infection spread by coughing and sneezing of infected people

1. Yes
2. No

Q17 Main symptoms that may be caused by COVID-19?

1. Fever
2. Cough
3. Breathlessness
4. Myalgia
5. Headache
6. Diarrhea
7. Fatigue

Q18 There are no antiviral drugs that are effective against COVID-19.

1. Yes
2. No
3. Do not know

Q19 There are no vaccine against COVID-19.

1. Yes
2. No
3. Do not know

Q20 What I think is the right way to prevent COVID-19.

1. Try to avoid mosquito bites
2. Hand wash using soap and running water
3. Wear a mask where there are people
4. Avoid close contact with anyone who has a fever
5. Ventilate the room
6. Stay in the room as much as possible
7. Eat well balanced meals
8. Eat meals alone
9. Sleep well

Q21 How threatening is COVID-19?

1. Very threatening
2. Threatening
3. Not so threatening

Q22 Factors that make COVID-19 feel threatening.

1. I might become infected myself
2. I might infect others
3. I might die
4. If I get infected, I might be quarantined
5. My work and life will be restricted
6. There is no effective medicine or vaccine

Q23 I will feel embarrassed if I become infected with COVID-19.

1. Yes
2. No

Q24 What was the issue that affect your preventable behavior?.

1. Government state of emergency
2. WHO Pandemic Declaration

3. Celebrity deaths from COVID-19 infection
4. Infection of close person
5. Death of close person due to COVID-19
6. Media news about the infection for famous people

Q25 I think that, in the near future, the situation of COVID-19 will converge.
1. Agree
2. Disagree
3. Neither agree/disagree

Q26 What if you or your family have symptoms that suggest COVID-19 infection?
1. Seek medical attention immediately
2. Call the health center for advice
3. Contact the workplace
4. Talk to family
5. Talk to a friend or colleague
6. Consult the family doctor
7. Go to the pharmacy and consult a pharmacist
8. Do not tell anyone
9. Do not go out of the house

Q27 Infection measures I have in place for COVID-19
1. Wear a mask where there are other people
2. Hand hygiene
3. Gargle
4. Ventilate the room regularly
5. Room cleaning and disinfection
6. Stay home on holidays
7. Avoid contact with people as much as possible
8. Eat well balanced meals
9. Sleep well
10. Do nothing

Q28 The infection control measures in the workplace where I work are sufficient.
1. Agree
2. Disagree

3. Neither agree/disagree

Q29 Infection control measures implemented in the workplace.

1. Disinfect indoor items and equipment
2. Ventilate the room regularly
3. Alert and educate patients about COVID-19

Q30 I want to keep my current job despite the high risk of COVID-19 infection.

1. Yes
2. No

Q31 I am proud of my job.

1. Yes
2. No

Q32 COVID-19 is a preventable disease.

1. Agree
2. Disagree
3. Neither agree/disagree

Q33 Influenza is a preventable disease.

1. Agree
2. Disagree
3. Neither agree/disagree

Q34 Select only one of the most scary infections

1. COVID-19
2. influenza
3. cold
4. tuberculosis
5. HIV
6. Dengue fever
7. MERS
8. SERS
9. Zika fever
10. tetanus

11. measles
12. rubella
13. Ebola
14. rabies
