# Supplementary figures and images for: Knowledge, Attitudes, and Practices Survey among Nursing Care Workers Involved in Caring for Older Adults during the Early Stage of the COVID-19 Pandemic in Japan
Source: Int J Environ Res Public Health. 2022 Oct 11;19(20):12993. doi: 10.3390/ijerph192012993 (PMC9602712; doi:10.3390/ijerph192012993)

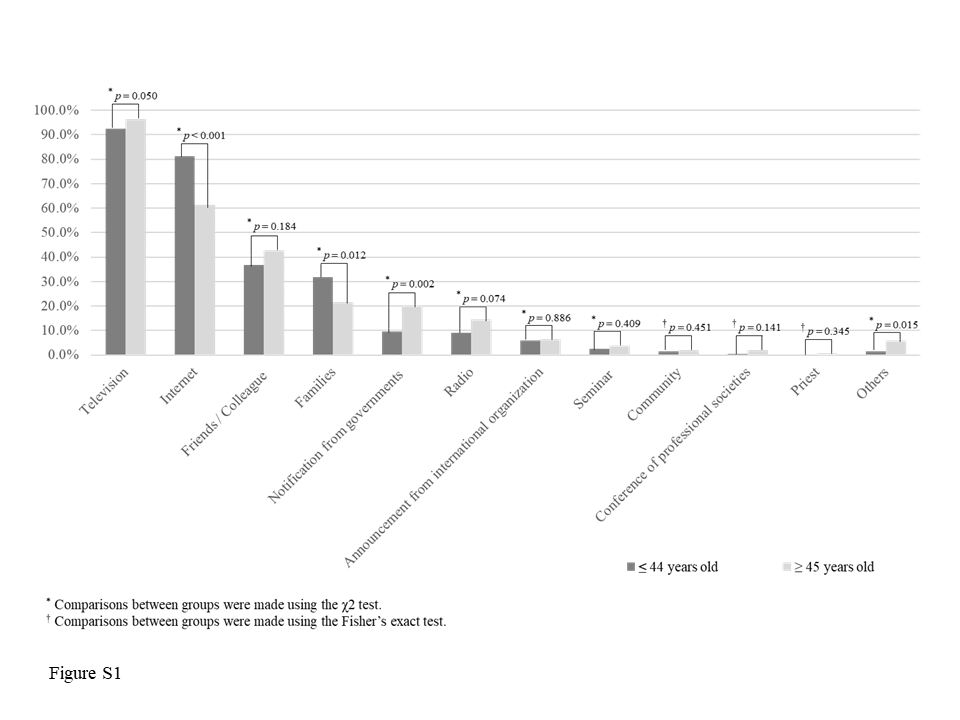

Supplement: Supplementary file 1 [file ijerph-19-12993-s001.zip › Figure S1.tif]

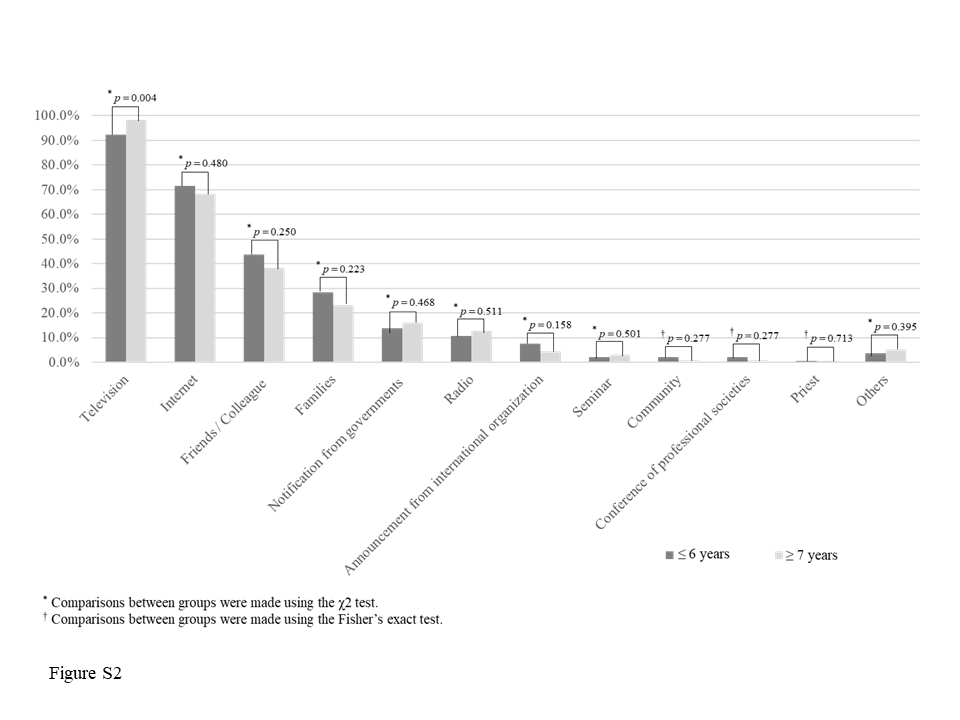

Supplement: Supplementary file 1 [file ijerph-19-12993-s001.zip › Figure S2.tif]
